# Distinct psychological mechanisms for explicit and implicit aggression: evidence from the narcissism and sense of power

**DOI:** 10.3389/fpsyg.2025.1519718

**Published:** 2025-04-02

**Authors:** Qi Hu, Zijun Meng, Jiantao Lu, Yu Zhu, Kaihua Zhang, Qian Zhang

**Affiliations:** ^1^School of Marxism, Shandong Normal University, Jinan, Shandong, China; ^2^School of Psychology, Shandong Normal University, Jinan, Shandong, China; ^3^School Hospital, Qilu University of Technology (Shandong Academy of Sciences), Jinan, Shandong, China; ^4^Zhongjing Experimental School, Tai’an, Shandong, China

**Keywords:** explicit aggression, implicit aggression, overt narcissism, covert narcissism, sense of power

## Abstract

**Introduction:**

Aggression has been demonstrated a distinction between explicit and implicit social cognition. Explicit aggression refers to the conscious tendency to display aggressive behavior while maintaining necessary self-control. Implicit aggression is an unconscious aggressive behavior shaped by past experiences that operates beyond an individual’s control. Although aggression comprises explicit and implicit structures, the psychological mechanisms of such dual aggression remain unclear. According to the general aggression model, when individuals are unable to balance the internal state (e.g., narcissism and power), aggression may occur. Therefore, the current study explored the psychological mechanisms of the dual aggression by using narcissism and power.

**Methods:**

Study 1 recruited 331 undergraduate students to complete online questionnaires assessing overt and covert narcissism, sense of power, and explicit aggression. A mediation model was constructed to examine the relationship among these variables. Furthermore, Study 2 assessed implicit aggression using the Implicit Association Test (IAT) to examine the relationship among narcissism, sense of power, and implicit aggression.

**Results:**

The results indicated that (1) covert narcissism was positively associated with explicit aggression, which was partially mediated by sense of power; (2) overt narcissism was positively correlated with explicit aggression, which was not mediated by sense of power; (3) overt and covert narcissism were positively associated with implicit aggression, whereas sense of power was not significantly correlated with implicit aggression; (4) there was no correlation between explicit aggression and implicit aggression.

**Discussion:**

These findings offer valuable insights into the distinct psychological mechanisms underlying the explicit and implicit aggression, as suggested that their predictors were distinct. Covert narcissism may interact with other factors (e.g., sensitivity and hostility) to activate sense of power, thereby eliciting explicit aggression. Whereas individuals with higher overt and covert narcissism showed stronger implicit aggression.

## 1 Introduction

Aggression refers to the deliberate act of causing physical or psychological harm to others (Anderson and Bushman, 2002). Previous research has demonstrated a significant experimental distinction between explicit and implicit social cognition regarding individuals’ aggressive behavior (Gollwitzer et al., 2007; Yang and Liu, 1996). Explicit aggression refers to an individual’s conscious tendency to display aggressive behavior while maintaining necessary self-control (Grumm et al., 2011). Implicit aggression is an unconscious structure shaped by past experiences that operates beyond an individual’s control and automatically influences their perceptions, attitudes, judgments, decisions, and reactions related to aggressive behavior (Todorov and Bargh, 2002; Uhlmann and Swanson, 2004), which is inherent in individuals (Ireland and Birch, 2013). The dual aggression model assumes that individual aggression comprises both explicit and implicit structures (Zhou and Liu, 2009). Nevertheless, few studies have successfully developed distinct prediction models for implicit and explicit aggression, and these models need additional verification.

Different methods are used to measure explicit and implicit aggression. Explicit aggression is assessed by questionnaires, while implicit aggression is measured with indirect measurement tools (Predoiu et al., 2024a,b). Explicit aggression is typically assessed through self-report methodologies, and encompasses physical aggression, verbal aggression, anger, and hostility dimensions (Buss and Perry, 1992). In contrast, implicit aggression is measured indirectly using reaction-time paradigms which primarily examines the degree of automatic association between concept words and attribute words through a classification task (Baumann et al., 2020). Empirical findings demonstrate that the self-reported dimensions of explicit aggression (including physical aggression, verbal aggression, anger, and hostility) show no significant correlation with reaction-time-based measurements of implicit aggression, suggesting that explicit and implicit forms of aggression are distinct psychological structures (Zhang and Wang, 2005). However, the methods of implicit and explicit measurement remain a subject of ongoing debate. One theory posits that implicit and explicit measurements capture distinct internal cognitive structures. Explicit structure is considered the outcome of conscious thought and self-reflection, whereas implicit structures arise from unconscious processes (Dovidio et al., 2002; McConnell and Leibold, 2001). Other theory argues that implicit and explicit measures reflect different stages of cognitive structure. Implicit measures assess pre-conscious representations, while explicit measures capture later stages influenced by conscious control (Fazio and Olson, 2003).

The mechanism of aggressive behavior is complex and diverse, which is related to physiological states (e.g., gender and age; Bosson, 2025; Nwufo et al., 2023; Peng et al., 2022) and social factors (e.g., culture, education level and family; Legkauskas, 2023; Wu et al., 2020). It has been also demonstrated that narcissism is correlated with both explicit and implicit aggression (Kjaervik and Bushman, 2021; Liu et al., 2024). Narcissism is a subject of great interest to both behavioral scientists and the general public (Miller et al., 2021). Researchers early defined narcissism from the perspectives of psychopathy and clinical science, and believed that narcissism is a pathological personality disorder (American Psychiatric Association [APA], 2013). In recent years, researchers explore narcissism from a non-clinical perspective. Narcissism is defined as “entitled self-importance” (Krizan and Herlache, 2018). Individuals with high level of narcissism tend to think that they are special and deserve special treatment (Kjaervik and Bushman, 2021). Researchers have proposed two distinct forms of narcissism: overt narcissism, which is characterized by conspicuous displays through which an individual seeks admiration and attention from others; and covert narcissism, which is marked by heightened sensitivity, low self-esteem, anxiety, and pronounced self-deception (Miller et al., 2011; Wink, 1991). Twenge and Campbell (2003) have examined the relationship between overt narcissism (particularly dimensions of power, superiority, and self-admiration) and explicit aggression, and found that individuals with heightened overt narcissism exhibited increased explicit aggression when experiencing social rejection. Subsequent study of narcissism subtypes revealed that grandiose narcissism (i.e., overt narcissism) was positively correlated with the dimensions of physical and verbal aggression, whereas vulnerable narcissism (i.e., covert narcissism) was positively correlated with internalized affective responses such as anger and hostility (Okada, 2010). Furthermore, covert narcissism predicted explicit and implicit aggression better than overt narcissism (Liu et al., 2021; Rasmussen, 2016). Individuals exhibiting higher levels of narcissism are predisposed to engage in aggressive behavior when faced with provocation (Kjaervik and Bushman, 2021). Previous research has preliminary revealed the correlation between (overt or covert) narcissism and (explicit or implicit) aggression. However, it remains unclear whether this relationship modulated by explicit and implicit social cognition for aggression. Therefore, we examined the influence of overt and covert narcissism on explicit and implicit aggression in college students to reveal the psychological mechanisms of aggression.

Furthermore, there has been a growing emphasis on investigating individual internal states within the context of aggressive behavior, particularly with respect to power (Volk et al., 2022; Weick, 2020). The general aggression model posits that an individual’s internal state significantly influences their aggressive behavior. Power represents a crucial factor influencing individual psychological and social interaction behaviors (Keltner et al., 2003; Anderson et al., 2012). The sense of power is a psychological attribute related to an individual’s perception of their ability to influence others and can be activated by hints or memories (Galinsky et al., 2003; Anderson et al., 2012). When individuals perceive themselves as having lower sense of power relative to others, they are more likely to engage in aggressive behavior (Warburton et al., 2006; Greitemeyer and Sagioglou, 2016; Weick, 2020). Research on the relationship between adolescents’ sense of power and aggression has shown that individuals with a low sense of power tend to exhibit explicit and implicit aggression in groups (Volk et al., 2022). Exploring the relationship between a sense of power and aggressive behavior in intimate relationships also reveals that when men have a lower sense of power, they are more likely to show explicit aggression toward their partners (Babcock et al., 1993; Harrington et al., 2021; Overall et al., 2016). Furthermore, narcissistic individuals often seek out positions of authority (Carroll, 1987; Joubert, 1998; Vrabel et al., 2020). Researchers utilized the NPI and the General Sense of Power Scale (GSPS) to reveal a positive correlation between the level of narcissism and sense of power, particularly in relation to power desire, superiority, and self-admiration (Anderson et al., 2012; Hawk et al., 2015). Taken together, although researchers have probed into the relationship between explicit and implicit narcissism as well as implicit and explicit aggression (Kjaervik and Bushman, 2021; Okada, 2010), few studies have explored and explained the relationship between the dual structures of narcissism and aggression simultaneously. Further, how overt and covert narcissism and sense of power impact an individual’s aggression remains unclear, especially from the perspective of explicit and implicit social cognition for aggression.

With respect to the transition from late adolescence to adulthood, college students represent a distinct demographic undergoing crucial physical and psychological development, whose levels of narcissistic personality traits and sense of power could variably impact both explicit and implicit forms of aggressive behavior (Barnett and Powell, 2016; Lee and Kang, 2020). Therefore, we recruited college students to investigate the impact of overt and covert narcissism and sense of power on explicit aggression (Study 1) and implicit aggression (Study 2). Considering that overt narcissism is typically characterized by outward displays and pretense, whereas covert narcissism manifests as extreme sensitivity, low self-confidence, and anxiety, overt and covert narcissism may have different effects on explicit and implicit aggression through the sense of power. Thus, we hypothesized that sense of power might mediate the relationship between narcissism (especially covert narcissism) and explicit aggression, whereas this mediating effect was not observed for implicit aggression. If explicit aggression and implicit aggression have distinct psychological mechanisms, their predictors would be distinct, thereby no correlation between explicit aggression and implicit aggression.

## 2 Study 1: the influence of overt and covert narcissism and sense of power on explicit aggression in college students

### 2.1 Methods

#### 2.1.1 Participants

We recruited 370 students from the university community through online surveys from January to March in 2020. Samples were excluded due to the completion time less than 1.5 min or failing more than half of the validity items, remaining 331 students (242 females, age range, 18–26 years, mean age: 21.88 years, *SD* = 1.41). Study 1 obtained approval from the Ethics Committee of the School of Psychology, Shandong Normal University.

#### 2.1.2 Measures

##### 2.1.2.1 Narcissistic Personality Questionnaire

The narcissistic personality questionnaire (NPQ) was used to measure overt and covert narcissistic, which was originally compiled by Raskin and Terry (1988). We used a Chinese version of the NPQ revised by Zheng and Huang (2005). The questionnaire consisted of 28 items and had two subscales including the overt narcissistic personality subscale (e.g., I really want to become a leader) and the covert narcissistic personality subscale (e.g., People often disappoint me). All items were scored on a five-point scale ranging from 1 (strongly disagree) to 5 (strongly agree). The score of all items was calculated, with higher scores indicating higher levels of overt and covert narcissism. The overt narcissistic (Cronbach’s *α* = 0.90) and covert narcissistic (Cronbach’s *α* = 0.82) results showed good internal consistency in this study.

##### 2.1.2.2 Generalized Sense Of Power Scale

Trait power was assessed using the Generalized Sense of Power Scale compiled by Anderson and Galinsky (2006), which measures a stable and enduring sense of power. The scale comprised 8 items, in which 4 items (Items 1, 3, 5, 8) were scored in forward (e.g., I can get others to do what I want) and 4 items (Items 2, 4, 6, 7) were scored in reverse (e.g., My wishes do not carry much weight). All items were scored on a seven-point scale ranging from 1 (strongly disagree) to 7 (strongly agree). The score of all items was calculated, with higher scores indicating higher levels of sense of power. The measurement results showed good internal consistency in this study (Cronbach’s *α* = 0.82).

##### 2.1.2.3 BUSS-Perry Aggression Scale

The BUSS-Perry Aggression scale complied by Buss and Perry (1992) and revised by Lv et al. (2013) was selected to measure explicit aggression, which consisted of 22 items. The questionnaire had four dimensions including hostility (e.g., I am sometimes eaten up with jealousy), physical aggression (e.g., I have threatened people I know), impulse (e.g., My friends say that I’m somewhat argumentative), and irritability (e.g., I sometimes feel like a powder keg ready to explode). All items were scored on a seven-point scale ranging from 1 (strongly disagree) to 7 (strongly agree). The score of all items was calculated, with higher scores indicating stronger explicit aggression tendencies. The measurement results showed good internal consistency in this study (Cronbach’s *α* = 0.89).

#### 2.1.3 Research procedure and data processing

Participants were required to spend approximately 15 min completing the self-report questionnaire online. They participated voluntarily and could withdraw at any time.

The descriptive statistical analysis of the variables was conducted in SPSS 21.0. Mediation effect was estimated using the PROCESS macro developed by Hayes (2013) implemented in SPSS 21.0. The significance of regression coefficients was tested using Bootstrapping procedure (with 5,000 bootstrap samples) which estimated 95% confidence intervals (CI). It is a significant indirect effect when the 95% CI does not include zero.

### 2.2 Results

#### 2.2.1 Common method bias test

The Harman single-factor analysis was performed on all items of the scales. The results revealed that the maximum factor explaining the variance was 18.17%, which was below the threshold of 40%. It indicates that there is no significant common method bias.

#### 2.2.2 Gender difference analysis

Independent samples *t*-tests were conducted to examine the gender difference among main variables: overt narcissism, covert narcissism, sense of power and explicit aggression (Table 1). The results indicated that the significant difference between males and females was only observed in overt narcissism, *t* (329) = 3.93, *p* < 0.001. The males’ overt narcissism score was greater than that of the females. Therefore, gender was used as the control variable in the subsequent analysis.

**Table 1:** Descriptive statistics for each observed variable.

Variables	Male	Female	Total score
Overt Narcissism	63.62 ± 12.08	57.91 ± 11.60	59.44 ± 11.98
Covert Narcissism	39.93 ± 9.70	38.59 ± 8.07	38.95 ± 8.55
Sense of Power	37.63 ± 7.28	36.17 ± 6.51	36.56 ± 6.75
Explicit Aggression	55.56 ± 13.48	52.43 ± 13.45	53.27 ± 13.51

#### 2.2.3 Correlation analysis

Table 2 shows correlation coefficients of the main variables. The results indicated that explicit aggression was positively correlated with both overt and covert narcissism, whereas was negatively correlated with sense of power. Sense of power was positively correlated with overt narcissism and negatively correlated with covert narcissism.

**Table 2:** Correlation analysis of overt and covert narcissism, sense of power and explicit aggression.

	1	2	3	4
1. Overt Narcissism	–			
2. Covert Narcissism	0.52^***^	-		
3. Sense of Power	0.39^***^	−0.20^***^	–	
4. Explicit Aggression	0.13^*^	0.50^***^	−0.29^***^	–

*p < 0.05; **p < 0.01; ***p < 0.001.

#### 2.2.4 Regression analysis

Explicit aggression served as the dependent variable. Overt and covert narcissism and sense of power were designated as independent variables. Gender was treated as the control variable. Multiple hierarchical regression analysis was shown in Tables 3, 4. The results revealed that overt narcissism significantly positively predicted explicit aggression (*β* = 0.27, *p* < 0.001), whereas sense of power significantly negatively predicted explicit aggression (*β* = −0.40, *p* < 0.001). Covert narcissism and sense of power collectively accounted for 13.50% of the total variance in explicit aggression.

**Table 3:** Regression analysis of overt narcissism, sense of power and explicit aggression.

	Step one		Step two	
	*β*	*t*	*β*	*t*
Gender	−0.10	−1.88	−0.09	−1.64
Overt Narcissism	–	–	0.27	4.76^***^
Sense of Power	–	–	−0.40	−7.26^***^
*F*	3.54		20.49^***^	
*R* ^2^	0.01		0.16	
△*R*^2^	0.01		0.14	

***p < 0.001.

**Table 4:** Regression analysis of covert narcissism, sense of power and explicit aggression.

	Step One		Step Two	
	*β*	*t*	*β*	*t*
Gender	−0.10	−1.88	−0.09	−1.95
Covert Narcissism	–	–	0.46	9.61^***^
Sense of Power	–	–	−0.21	−4.32^***^
*F*	3.54		46.34^***^	
*R* ^2^	0.01		0.30	
△*R*^2^	0.01		0.04	

***p < 0.001.

Covert narcissism significantly positively predicted explicit aggression (*β* = 0.46, *p* < 0.001), and sense of power significantly negatively predicted explicit aggression (*β* = −0.21, *p* < 0.001) after controlling gender. Together, covert narcissism and sense of power accounted for 4.00% of the total variance in explicit aggression.

#### 2.2.5 Mediation analysis

**Figure 1:**
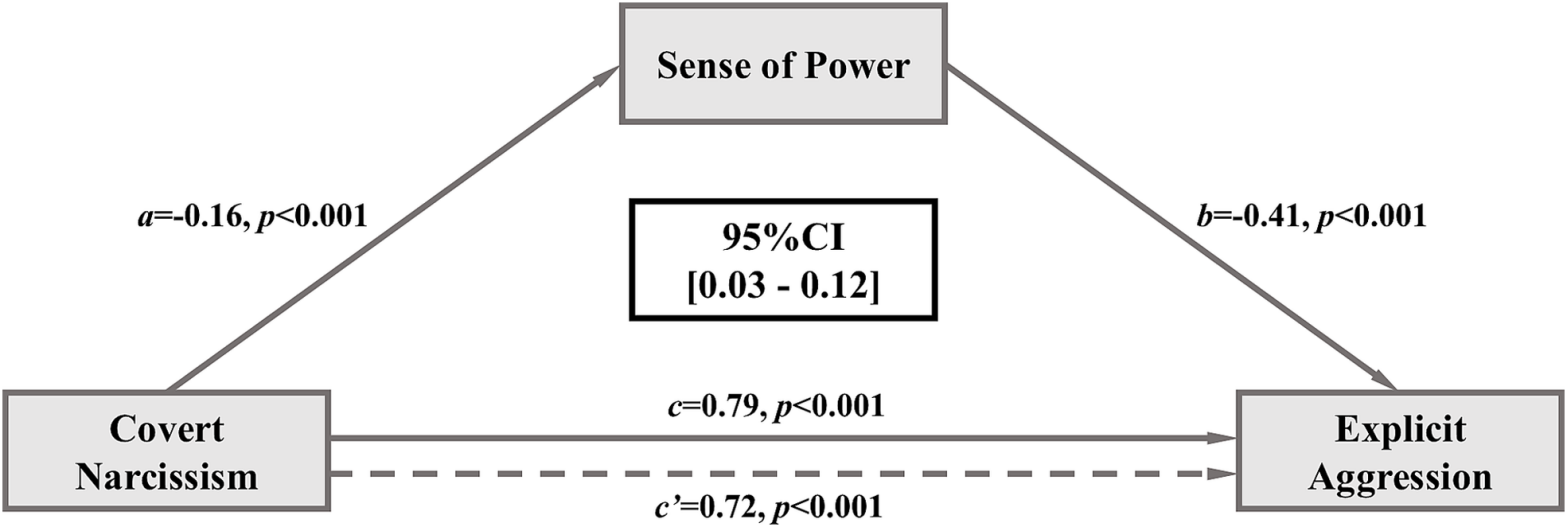
Mediation analyses. Given that in both cases c’ is also statistically different from zero, they support partial mediation.

To further investigate the influence of sense of power on the relationship between explicit aggression and covert narcissism, we employed Model 4 from the PROCESS for SPSS proposed by Hayes (2013) to analyze the mediation effect (Figure 1). All variables were standardized. The analysis revealed a significant mediation effect of sense of power, with a 95% CI of 0.03–0.12. Specific values of indirect and direct effects of covert narcissism on explicit aggression are shown in Table 5. The mediating effect of sense of power accounted for 8.86% of the total effect. It suggested significant associations between explicit aggression and covert narcissism, partially through sense of power.

**Table 5:** Direct and indirect effects of covert narcissism on explicit aggression.

Mediator		Effect value	Boot standard error	95%CI	Relative effect value
Sense of power	Total effect	0.79			
	Direct effect	0.72	0.08	[0.57, 0.87]	91.14%
	Indirect effect	0.07	0.02	[0.03, 0.12]	8.86%

## 3 Study 2: the influence of overt and covert narcissism and sense of power on implicit aggression in college students

### 3.1 Method

#### 3.1.1 Participants

Fifty-nine participants (42 females; age range: 18–24 years, mean age: 21.15 years, *SD* = 1.92) participated in this study from January to March in 2020. All participants in the study were selected from Study 1. They were right-handed and had normal or corrected-to-normal vision. Study 2 obtained approval from the Ethics Committee of the School of Psychology, Shandong Normal University.

#### 3.1.2 Measures

The measures were the same as those in Study 1. In Study 2, both overt narcissistic (Cronbach’s *α* = 0.77) and covert narcissistic (Cronbach’s *α* = 0.79) results showed good internal consistency in this study. The Generalized Sense of Power Scale (Cronbach’s *α* = 0.72) and the BUSS - Perry Aggression Scale (Cronbach’s *α* = 0.85) results also showed good internal consistency in this study.

#### 3.1.3 Materials

The stimuli were category and attribute words, which were selected from previous research (Dai et al., 2006; Zhang et al., 2009). The category words included six ego words (I/我/俺/本人, we/咱/我们/咱们) and six non-ego words (you/你, she/她, he/他, others/他人/别人, them/他们). The attribute words included six aggressive words (brawl/斗殴, punch/拳打, violate/侵犯, fight /搏斗, attack /攻击, grab/抢夺) and six nonaggressive words (love/友爱, calm/安宁, peace/和平, gentle/温和, trust/信任, soft/温柔). A pilot experiment was conducted to test the degree of implicit aggression for the attribute words. Each participant rated their degree of implicit aggression with each category word using a 10-point rating scale. The result showed a significant difference in the perception of implicit aggression between aggressive and nonaggressive words, *t*(19) = 24.11, *p* < 0.001, see Supplementary Tables S1, S2.

Each word subtended an area of 1° × 1° of visual angle on a computer screen. All the words were displayed with E-Prime 2.0 software (Psychology Software Tools Inc., Sharpsburg, PA, USA) on a white background projected onto an LCD screen with a resolution of 1,024 × 768 pixels. The viewing distance between the participants’ eyes and the screen was approximately 57 cm. Other measure aspects were the same as in Study 1.

#### 3.1.4 Procedure

IAT measures automatic associations in memory and is a reaction time task that reflects strength of associations between difference concepts. The IAT procedure was based on the study of Baumann et al. (2020). In this study, the task comprised 5 blocks (Table 6). Participants completed 132 trials. The task lasted approximately 10 min.

**Table 6:** IAT program on implicit aggression.

Block	Task name	Trials	*F* Key	*J* Key
1	Target Word Discrimination	12	ego	non-ego
2	Attribute word Discrimination	12	non-aggression	aggression
3	Joint Task 1	48	ego + non-aggression	non-ego + aggression
4	Reverse Target Word	12	non-ego	ego
5	Joint Task 2	48	non-ego + non-aggression	ego + aggression

Joint task 1 is the incompatible task and joint task 2 was the compatible task.

#### 3.1.5 Data processing

Following the data screening criteria proposed by Greenwald et al. (2003), participants with an error rate exceeding 20% were excluded (error rates of all data <20% in this study); subsequently, the reaction times of joint task 1 and joint task 2 were reviewed, with reaction time longer than 3 s recorded as 3 s and those shorter than 0.3 s recorded as 0.3 s.

To evaluate the implicit aggression, the implicit aggression effect called *D* value were measured and analyzed, which reflected the sensitivity index of implicit attitude difference. *D* value was the reaction times of incompatible task minus the reaction times of compatible task, in which incompatible task was the joint task I (i.e., non-ego + aggression and ego + non-aggression) and compatible task was the joint task II (i.e., ego + aggression and non-ego + non-aggression). The higher *D* value, the greater the implicit aggression (Baumann et al., 2020).

### 3.2 Results

#### 3.2.1 Common method bias test

The Harman single-factor analysis revealed that the maximum factor explaining the variance was 17.34%, which was below the threshold of 40%. It suggests that there is no significant common method bias in the Study 2.

#### 3.2.2 Gender difference analysis

According to the independent-samples *t* test (Table 7), males had higher covert narcissism and explicit aggression scores than females, *t_1_*(57) = 2.14, *p_1_* = 0.037; *t_2_*(57) = 2.89, *p_2_* = 0.006. The overt narcissism score of males was marginally higher than females, *t*(58) = 1.98, *p* = 0.053. No significant differences between males and females in sense of power and implicit aggression were observed (*ps* > 0.05). Therefore, gender was used as the control variable in the subsequent analysis.

**Table 7:** Descriptive statistics for each observed variable.

Variables	Male	Female	Total score
Overt Narcissism	63.94 ± 5.62	59.50 ± 8.52	60.78 ± 8.01
Covert Narcissism	41.00 ± 8.99	36.36 ± 6.90	37.69 ± 7.78
Sense of Power	36.82 ± 5.82	38.86 ± 5.57	38.27 ± 5.67
Explicit Aggression	57.29 ± 13.05	48.00 ± 10.40	50.68 ± 11.89
Implicit Aggression	−0.21 ± 0.26	−0.33 ± 0.31	−0.30 ± 0.30

#### 3.2.3 Correlation analysis

The results indicated that implicit aggression was positively correlated with both overt and covert narcissism (Table 8). Sense of power was negatively correlated with covert narcissism, whereas was not significantly correlated with both overt narcissism and implicit aggression.

**Table 8:** Correlation analysis of overt and covert narcissism, sense of power and implicit aggression.

Variables	1	2	3	4
1. Overt Narcissism	–			
2. Covert Narcissism	0.44^***^	–		
3. Sense of Power	0.22	−0.40^**^	–	
4. Implicit Aggression	0.27^*^	0.26^*^	0.03	–

*p < 0.05; **p < 0.01; ***p < 0.001.

#### 3.2.4 Regression analysis

Implicit aggression served as the dependent variable. Overt and covert narcissism were designated as independent variables. Gender was treated as the control variable. Multiple hierarchical regression analysis was shown in Tables 9, 10. The results revealed that neither overt (*β* = 0.24, *p* = 0.08) nor covert narcissism (*β* = 0.22, *p* = 0.10) significantly predicted implicit aggression.

**Table 9:** Regression analysis of overt narcissism and implicit aggression.

	Step one		Step two	
	*β*	*t*	*β*	*t*
Gender	−0.19	−1.47	−0.13	−1.00
Overt Narcissism	–	–	0.24	1.78
*F*	2.16		2.71	
*R* ^2^	0.04		0.09	
△*R*^2^	0.04		0.05	

**Table 10:** Regression analysis of covert narcissism and implicit aggression.

	Step one		Step two	
	*β*	*t*	*β*	*t*
Gender	−0.19	−1.47	−0.13	−0.98
Covert Narcissism	–	–	0.22	1.68
*F*	2.16		2.52	
*R* ^2^	0.04		0.08	
△*R*^2^	0.04		0.05	

#### 3.2.5 Correlation analysis between explicit and implicit aggression

The evidence was insensitive for whether or not there was a significant correlation between explicit and implicit aggression, *r* = −0.113, *p* = 0.395.

## 4 General discussion

The current study aimed to explore the psychological mechanisms of the dual aggression by using overt and covert narcissism and sense of power in college students. Study 1 examined the interactions between overt and covert narcissism, sense of power, and explicit aggression. Study 2 examined the relationship between overt and covert narcissism, sense of power, and implicit aggression. Several meaningful findings were observed. Firstly, covert narcissism was positively associated with explicit aggression, which was partially mediated by sense of power; whereas although overt narcissism was positively correlated with explicit aggression, this correlation was not mediated by sense of power. Secondly, overt and covert narcissism were positively associated with implicit aggression, whereas sense of power was not correlated with implicit aggression. Finally, there was no correlation between explicit aggression and implicit aggression.

### 4.1 The association between overt and covert narcissism and explicit aggression: the role of sense of power

The findings of Study 1 indicated a significant positive correlation between explicit aggression and both covert and overt narcissism in college students, as well as a negative correlation between sense of power and explicit aggression. Covert narcissism and sense of power collectively accounted for changes in individual explicit aggression. An elevated level of covert narcissism correlates with an increased degree of explicit aggression, potentially because covert narcissism is a predictor of greater anger internalization and externalization, as well as diminished anger regulation (Krizan and Johar, 2015), which culminates in more aggressive behaviors. These findings are in line with previous studies (Okada, 2010; Liu et al., 2021). Moreover, sense of power negatively predicts individuals’ explicit aggression, which is consistent with the results of previous research (Babcock et al., 1993; Overall et al., 2016). When sense of power is perceived as being threatened, individuals engage in a series of behaviors aimed at protecting and restoring their sense of power, which often manifests as aggressive behavior (Salzano et al., 2023). Meanwhile, the low level of sense of power could activate the behavioral inhibition system in individuals, leading to the heightened sensitivity to threat cues and the increased experiences of negative emotions, which elevates the risk of aggressive behavior (Keltner et al., 2003).

Overt and covert narcissism was associated with sense of power in this study, which is consistent with findings of previous research (Anderson et al., 2012; Hawk et al., 2015). Specifically, sense of power has a positive correlation with overt narcissism, whereas has a negative correlation with covert narcissism. Compared with overt narcissists, covert narcissists are more prone to experiencing depression, anxiety, and sadness (Miller et al., 2011), which may result in a lower sense of power. In contrast, overt narcissists’ strong individualism and pursuit of power make them more conscious of their own sense of power.

Other than taking a pure correlational view regarding the role of overt and covert narcissism in explicit aggression, the present study tried to explain how it works, by testing whether sense of power could account for the association between overt and covert narcissism and explicit aggression. Our results showed that sense of power acted as a partial mediational mechanism linking covert narcissism and explicit aggression; however, the mediation effect of sense of power on the relationship between overt narcissism and explicit aggression was not significant. Compared with overt narcissists, covert narcissists are more prone to experiencing traumatic events and also more susceptible to being affected by negative emotions such as anxiety and depression (Krizan and Johar, 2015), which could result in a sustained low sense of power. In this state, individuals frequently employ explicit aggression as a strategy to sustain equilibrium and enhance their sense of power.

### 4.2 Overt and covert narcissism and implicit aggression

The findings of Study 2 indicated a positive correlation between overt and covert narcissism and implicit aggression. Both overt and covert narcissism were positively associated with implicit aggression. These results align with Okada (2010) study. Research has indicated that the level of covert narcissism may interact with sensitivity, hostility, and other factors to activate self-defense mechanisms and provoke implicit aggression (Fossati et al., 2010). Individuals with covert narcissism display extreme sensitivity, a lack of confidence, timidity, anxiety, and elaborate self-fantasies (Wink, 1991). While this self-illusion resembles the self-exaggeration seen in overt narcissists, the fragility of covert narcissists leads them to unconsciously employ aggressive means to protect themselves after prolonged experiences of negative emotions. As a result, their aggression is automatic and implicitly expressed.

We did not observe the significant correlation between sense of power and implicit aggression. This may be because, in contrast to explicit aggression, implicit aggression represents an unconscious structure derived from past experiences, which operates beyond individual control and could automatically influence perceptions, attitudes, judgments, decisions, and reactions related to aggressive behavior (Todorov and Bargh, 2002; Uhlmann and Swanson, 2004). Consequently, the association between sense of power and implicit aggression is not as pronounced as that between sense of power and explicit aggression.

It is worth noting that there is no significant correlation between explicit aggression and implicit aggression, thus providing support for the dual aggression model. This model posits that questionnaire-based measures of aggression and those derived from implicit cognitive methods represent distinct psychological structures. Implicit aggression influences an individual’s spontaneous responses, which may be beyond their control or awareness, whereas explicit aggression impacts conscious behavior and could be consciously regulated (Yang and Liu, 1996; Yang et al., 1997; Dai et al., 2006; Zhou and Liu, 2009). These findings indicate that explicit and implicit aggression are two relatively independent psychological structures.

Moreover, although the results of Study 2 reveal the independent psychological structures of the dual aggression, it is based on a relatively small sample size. This leads us to be careful when making inferences. For the implicit measures in reflecting participants’ true conditions, recent studies suggest that self-reports serve as more reliable, predictive, and flexible measurement instruments compared to implicit measures (Corneille and Gawronski, 2024). Future studies should consider the implicit personality-related measures to measure implicit aggression.

### 4.3 Limitations

Several limitations that should be considered in future studies. Firstly, based on the data collection of cross-sectional design, such data can only reveal the relationship between variables, and is difficult to build a time series causal model. Future studies could enhance the causal explanation through cross-lagged panel design or randomized controlled experiments. Secondly, although the present study adopts the common research method for the implicit measures, the latest research shows that self-report is a better research method than implicit measurement. The implicit personality-related measures should be considered in future studies. Thirdly, this study focused on Chinese undergraduate students. This limited the generalizability of findings to broader age groups or cultural contexts. Future studies should include participants from different age groups, regions and cultural backgrounds to improve the external validity and generalizability of the research. Finally, potential variables, such as social dominance and status-seeking behavior, could affect the psychological mechanisms of the dual aggression. Future studies should consider these potential variables to provide a more comprehensive picture.

### 4.4 Practical implications

The current study explores the psychological mechanisms of the dual aggression by using overt and covert narcissism and sense of power in college students and obtained meaningful results. Our findings suggest several important insights from a standpoint of practical education. Firstly, college students are at a critical stage of psychological development, including the formation of identity and the establishment of interpersonal relationships. They inevitably encounter explicit and implicit aggression, which may be verbal aggression, cyber violence or relationship aggression. Educators should be attentive their mental health and provide appropriate support and intervention strategies to help students cope with aggression effectively. This may involve narcissism cognitive adjustment, power restructuring and responsibility education. Secondly, few college students are aware of how narcissism and sense of power affect explicit and implicit aggression. Mental health educators can provide mental health education courses and interpersonal relationship counseling to help college students enhance self-awareness and self-acceptance, cultivate empathy, and adjust the perception of power, thereby reducing aggression. Finally, this study provides a new perspective for the prevention and intervention in college students’ aggression. Educators should pay attention to the role of narcissism and sense of power when preventing and intervening aggressive behavior of college students.

## 5 Conclusion

The current study investigated the impact of overt and covert narcissism and sense of power on explicit aggression and implicit aggression using the IAT. The results showed that covert narcissism was positively associated with explicit aggression, which was partially mediated by sense of power; overt narcissism was positively correlated with explicit aggression, which was not mediated by sense of power. Overt and covert narcissism were positively associated with implicit aggression, whereas sense of power was not significantly correlated with implicit aggression. In addition, there was no correlation between explicit aggression and implicit aggression. These findings provide valuable insights into the psychological mechanisms underlying the explicit and implicit aggression, as suggested that their predictors were distinct.

## Data Availability

The datasets presented in this study can be found in online repositories. The names of the repository/repositories and accession number(s) can be found at: https://osf.io/spqtd/.
