# Supplementary material for: Distinct psychological mechanisms for explicit and implicit aggression: evidence from the narcissism and sense of power
Source: Front Psychol. 2025 Apr 2;16:1519718. doi: 10.3389/fpsyg.2025.1519718 (PMC12000008; doi:10.3389/fpsyg.2025.1519718)
Supplement: Supplementary file 1 [file Table_1.docx]

**Supplementary Material**

**TABLE S1** Degree of aggression rating of each aggressive word *(M* ± *SD)*

|  | brawl/斗殴 | fight/搏斗 | violate/侵犯 | attack/攻击 | punch/拳打 | grab/抢夺 |
| --- | --- | --- | --- | --- | --- | --- |
| aggressive words | 8.45±1.15 | 6.95±1.73 | 9.00±1.17 | 8.45±1.88 | 8.10±2.05 | 7.75±2.59 |

**TABLE S2** Degree of aggression rating of each nonaggressive word *(M* ± *SD)*

|  | peace/和平 | love/友爱 | gentle/温和 | trust/信任 | calm/安宁 | soft/温柔 |
| --- | --- | --- | --- | --- | --- | --- |
| nonaggressive words | 1.10±0.31 | 1.35±0.75 | 1.80±1.74 | 1.90±1.48 | 1.20±0.62 | 1.35±0.75 |
